# Analysis of Differentially Expressed Genes That Aggravate Metabolic Diseases in Depression

**DOI:** 10.3390/life11111203

**Published:** 2021-11-07

**Authors:** Sukanta Bhadra, Siyu Chen, Chang Liu

**Affiliations:** School of Life Science and Technology, China Pharmaceutical University, Nanjing 210009, China; 1879030243@stu.cpu.edu.cn (S.B.); siyuchen@cpu.edu.cn (S.C.)

**Keywords:** depression, metabolic disease, diabetes, obesity, NASH, DEGs

## Abstract

Depression is considered the second leading cause of the global health burden after cancer. It is recognized as the most common physiological disorder. It affects about 350 million people worldwide to a serious degree. The onset of depression, inadequate food intake, abnormal glycemic control and cognitive impairment have strong associations with various metabolic disorders which are mediated through alterations in diet and physical activities. The regulatory key factors among metabolic diseases and depression are poorly understood. To understand the molecular mechanisms of the dysregulation of genes affected in depressive disorder, we employed an analytical, quantitative framework for depression and related metabolic diseases. In this study, we examined datasets containing patients with depression, obesity, diabetes and NASH. After normalizing batch effects to minimize the heterogeneity of all the datasets, we found differentially expressed genes (DEGs) common to all the datasets. We identified significantly associated enrichment pathways, ontology pathways, protein–protein cluster networks and gene–disease associations among the co-expressed genes co-expressed in depression and the metabolic disorders. Our study suggested potentially active signaling pathways and co-expressed gene sets which may play key roles in crosstalk between metabolic diseases and depression.

## 1. Introduction

Major depressive disorder (MDD) is a psychiatric disease. It is considered one of the major global health burdens [1]. An estimation by the WHO suggested that 350 million people of all ages suffer from MDD that involves serious suicidal ideation [2,3]. External stressors can act as stimuli that cause MDD, depending on their repetitiveness and durations [4]. The pathophysiological responses in MDD include adaptive hormonal changes, such as increases in corticosteroids and adrenocorticotropin, which altogether play a significant role in the hyperactivity of the hypothalamic–pituitary–adrenal (HPA) axis at the onset of depression [5]. Studies over the years have found that there is a significant association between obesity and depression [6]. The excess intake of macronutrients above physiological requirements raises the sugar and the fat depots by increasing body weight, which promotes obesity. Obesity has been associated with many metabolic dysfunctions which in turn may act as stimulators of MDD [7]. Metabolic disorders such as hyperlipidemia and hyperglycemia are prevalent in a wide range of the psychiatric patients with MDD [8]. However, a strong link between depression and obesity has not been established [6]. Reports suggest that prediabetic patients and undiagnosed diabetic patients have more prevalence of depression compared to nondiabetic individuals [9]. Patients with type 1 and type 2 diabetes tend to acquire much more clinical symptoms of depression than nondiabetic individuals [10]. Overactivation of innate immunity by the cytokine-mediated inflammatory response leads to dysregulation of the hypothalamic–pituitary–adrenal (HPA) axis [11]. Hyperactivation of the HPA axis can disrupt metabolic homeostasis and promote obesity. HPA axis hyperactivation can include corticotrphin releasing hormone (CRH) suppression and leptin resistance. Activation of the HPA axis and the sympathetic nervous system (SNS) increases the production of adrenalin, noradrenalin and cortisol, promoting insulin resistance, visceral obesity and diabetes. Simultaneously, proinflammatory cytokines might directly affect the brain, causing depressive symptoms [12]. Reports from meta-analyses also support that depression may increase the risk of developing type 2 diabetes [13]. Hyperphagia is a commonly observed symptom in patients with depressive disorders, and this may cause issues with hepatic biochemistry, including hepatic injury, elevated hepatic enzymes and loss of hepatic blood flow [14]. These histological abnormalities during depression may cause liver disease: NAFLD ranges from simple steatosis to NASH and fibrosis [15]. Reports showed that the prevalence of MDD is higher in the NASH population than that in control subjects [16]. Importantly, they found that the diagnosis of MDD tended to be associated with steatosis grades [17]. It is widely accepted that psychiatric disorders, including MDD, are associated with histological severity of liver diseases [17]. As it is known that depression is associated with inflammation [18], which in turn affects insulin resistance, which could be relevant to the causality of NASH [14]. The associations between depressive disorder and the above-mentioned metabolic diseases have been established, but the genetic co-expression and interconnections among them have not been well elucidated yet. We employed a systematic approach to study independent microarray datasets related to depression, obesity and related metabolic disorders—diabetes and NASH. To understand the effects of depression on these metabolic diseases, we studied differentially dysregulated genes, enriched pathways, ontology analysis and protein–protein interactions; and lastly, for validation of our study, we compared the results with another OMIM disease database and datasets related to depression.

## 2. Methods and Materials

### 2.1. Overview of the Study

To identify links between depression and its effects on metabolic disorders, we employed microarray datasets from NCBI. Differentially expressed genes (DEGs) identified in these datasets were further analyzed to find out the commonly expressed DEGs among depression and related metabolic diseases. These genes were further used to construct a gene–disease association network, analyze KEGG pathways, analyze ontological pathways (GO) and perform protein–protein-interaction (PPI) network analysis. To validate the potential DEGs identified from enrichment analysis, the OMIM disease database and dbGap were used.

### 2.2. Retrieval of Microarray Datasets

We analyzed gene expression data with 4 independent experimental microarray datasets—accession numbers GSE58430 [19], GSE128021 [20], GSE43950 [21] and GSE43600 [22]—from the National Center for Biotechnology Information (NCBI) GEO2R database, as shown in Table 1. Dataset GSE58430 was produced by using human transcriptomic profiling of peripheral blood CD4+ T-lymphocyte cells. There were four groups: nondepressive asthma, depressive asthma, depression and healthy controls. We compared depressed patients with the healthy controls. The obesity dataset GSE128021 was generated from gene expression differences in omental mesothelial cells from the lean and obese human donors with a cross-sectional case–control study with two different cohorts of lean and obese patients with varying different degrees of obesity. The diabetes dataset GSE43950 was produced by gene expression profiling in endothelial precursor cells of patients protected from microvascular complications by modulating the TGF-β/PAI-1 axis in CD34+ cells from diabetic patients and controls. The sample of the dataset was constructed with peripheral blood from healthy controls, and from patients with long-standing, poorly controlled diabetes with severe microvascular complications blood and CD34+ cells were obtained. RNA extraction was followed by AffyNugen amplification, and the cDNA was probed to the Human RSTA Affymetrix 2.0 chip. The NASH dataset GSE43600 was constructed with liver samples from patients with varying severities of steatosis. The summarized description of the datasets is shown in Table 1.

### 2.3. Differential Gene Expression Analysis and Validation with Random Unrelated Diseases

Each dataset was normalized by quantile normalization, and the R package limma was used to identify the DEGs between depression and related metabolic disease datasets. The batch effects from each dataset were removed by ComBat method [23]. To determine the upregulated and downregulated genes, the threshold values were set to log FC > 1 *p* value < 0.05. The Venn diagram was also constructed using the Venn Diagram package in R. All significant DEGs are presented in volcano plots generated using R software.

To validate the datasets adopted, we have compared selected diseases in the present study with an unrelated, random infectious disease, influenza, shown in Table 2. Interestingly, we found that below 2% of the upregulated and 1% of the downregulated genes were observed in the datasets for unrelated diseases. Altogether, these findings suggest that the observed common DEGs were not found by chance, but the relatedness of the diseases caused higher chances of their occurrence; hence the rationale of this study is to find the DEGs expressed commonly between depression and metabolic disorders.

### 2.4. Gene Set Enrichment Analysis

Pathway-based analysis reveals the molecular crosstalk in the progression of complex diseases networks. We analyzed pathways of the commonly altered DEGs in depression and related metabolic diseases datasets using Enrichr [24], a comprehensive web-based gene-set enrichment tool, to construct KEGG pathways. Enrich R implements the Fisher exact test, in which a binomial distribution and independence are assumed for the probability of any gene belonging to any set from the random input gene list, in order to create the mean rank and standard deviation from the expected rank. It is followed by the correction to the next Fisher exact test, which calculates a Z score for the standard deviation. This is considered as a new, corrected score. Alternatively, a *p*-value obtained from the Fisher exact test can be combined with the Z score of the deviation. Hence, we combined the *p*-value obtained from the Fisher exact test and combined with the z-score of the deviation from the expected rank by multiplying these two numbers as follows:

c = log(*p*)⋅zc = log(*p*)·z

where c is the combined score, *p* is the *p*-value computed using the Fisher exact test, and z is the z-score computed by assessing the deviation from the expected rank.

The clustering level z-scores and *p*-values are highlighted in red if the clustering is significant (*p*-value < 0.1). This clustering indicator provides an additional assessment of related genes and measures their relevance in the specific gene-set libraries from the input list of genes. The observation of one or two clusters on the grid suggests that a gene-set library is relevant to the input list. It also indicates that the terms in the clusters are relevant to the input list. We considered significant signaling pathways after applying several statistical analyses in R packages, such as fgsea and MSigDB to access the KEGG gene sets, and clusterprofiler where we used hypergeometric tests to find out the statistically enriched KEGG pathways. To prevent a high false-discovery rate (FDR < 0.05) in multiple testing, q values were also estimated. We performed gene-set enrichment analysis for KEGG pathways significantly associated with upregulated and downregulated DEGs which were identified from the employed datasets (*p* value < 0.05)

### 2.5. Ontology Pathway

To analyze the ontology pathway significantly, potential biological process (BP), molecular function (MF) and cellular components (CC) involved in overlapping DEGs among depression and other metabolic diseases, we used the online database Enrichr to conduct the ontology-pathway enrichment analysis.

### 2.6. Protein–Protein Interaction (PPI) Network and Hub-Gene Identification

The protein–protein interactions of overlapping DEGs with a combined score > 0.4 were identified with systematic approaches in the STRING database [25], and we mapped the protein functional associations and protein–protein interactions (PPI). We use Cytoscape software [26] (http://www.cytoscape.org/, (accessed on 20 June 2021), version 3.7.1; Institute for Systems Biology, Seattle, WA, USA software plugin) to visualize and construct the transcriptional regulatory network of common DEGs among the metabolic disease and depression database. After overlaying DEGs on networks in CytoHubba plugin in Cytoscape, hub genes with >10 degree were identified among all the modules from different datasets.

Molecular complex detection was used to find the most significant functional interactions between proteins’ modular clusters from dense PPI network regions [27]. Module identification criteria were included with a degree cut-off of 2, node score cut-off of 0.2, k- core of 2 and the maximum depth of 100. Significant modules were identified with MCODE score > 3 and nodes > 3. The cluster networks were visualized with Cytoscape 3.7.1 software plug-ins.

### 2.7. Prediction of the Master Transcription Factors (TFs)

To predict master TFs that significantly regulate the DEGs, we have utilized the iRegulon plugin of Cytoscape software (version 3.8.0) to detect regulons from all DEGs [28]. The iRegulon method is determined by a ranking-and-recovery system. In this method, all genes of the human genome are scored by a motif discovery step which connects the cluster binding sites within cis-regulatory modules (CRMs) and the potential distal location of CRMs of the transcription start site (TSS ± 10 kb) in both upstream and downstream. The normalized enrichment score (NES) is computed at the recovery step for TFs of each set of genes. The prediction of the TFs is based on NES and their putative target genes directly from the input lists. The association of TFs to motifs using both explicit annotations and predictions of TF orthologs and motif similarity is optimized by this method. A transcription factor normalized enrichment score was computed for each group, where a normalized enrichment score > 4.0 is considered significant, and the maximum false-discovery rate (FDR) for motif similarity was set as 0.001.

### 2.8. OMIM Disease and OMIM Expanded Database

In order to validate the results obtained from various analyses, we selected the OMIM database [29] and the dbGap benchmark database [30] to verify the significantly expressed genes from co-expression analysis in depression and other metabolic disease datasets.

### 2.9. Validation of the Common DEGs with other Depression-Related Datasets

To compare the DEGs commonly expressed on each disease-related dataset, we compared the expression in other datasets related to depression and depression-induced carcinoma. GSE 14922 [31] is produced by transcriptional profiling of human adrenocortical tumors in which we compared primary adrenocortical cancer tissues with the normal adrenal cortex. GSE109857 [32] is composed of WHO-classified grade-III glioma with the healthy controls. In GSE114852 [33], prenatal exposure to maternal stress and depression has been considered as the risk factor for depression, and we compared healthy mothers with those with depression. In GSE32280 [33], we compared the whole-genome microarray expression profile of leucocytes with SSD (somatic symptom disorder) and MDD patients.

### 2.10. Statistical Analysis

Statistical analysis was performed using the Origin 8 software (version 8.6, OriginLab Corporation, Massachusetts, MA, USA) and Graphpad Prism 8 (GraphPad, San Diego, CA, USA). The unpaired Student’s *t*-test (two-tailed) and one-way ANOVA followed by Fisher’s least significant difference (LSD) post hoc test were performed to analyze the data, where appropriate. A value of *p* < 0.05 was considered as statistically significant. N.S.: not significant (*p* > 0.05).

To avoid complications arising from the different experimental systems, compound traits or ethnicity used in the original datasets, we normalized the gene expression data by using Z-score transformation (Zij) for each type of tissue gene expression profile

Zij = (gij− mean g)/(SD(gi))

where SD represents the standard deviation, gij denotes the value of the gene expression i in sample j. Transformed gene expression values using the Z score of different diseases at different platforms can be compared according to a previous study [34].

## 3. Results

### 3.1. Identification of DEGs

**Figure 1:**
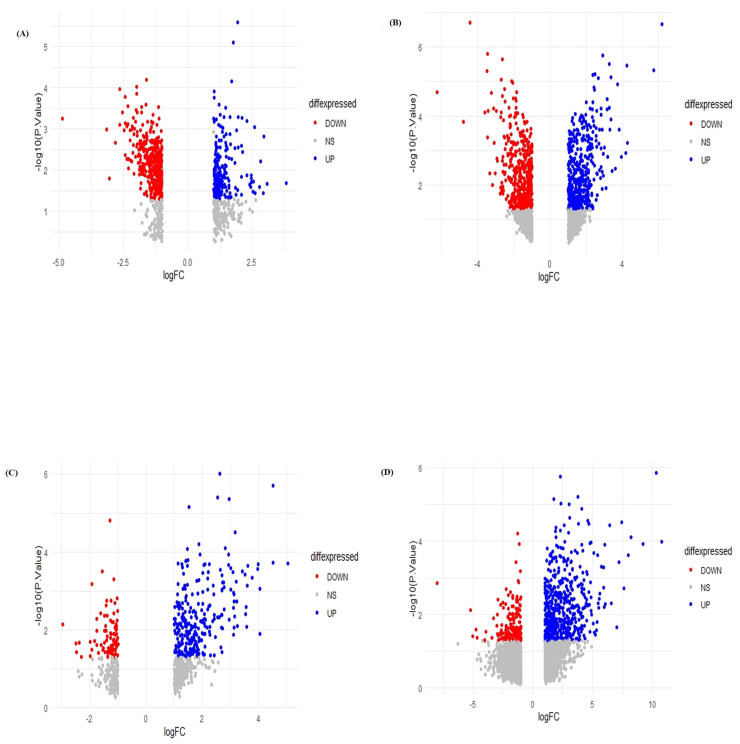
(**A**–**D**) volcano plots showing the significantly up- and down-regulated differentially expressed genes from depression, obesity, diabetes and NASH datasets.

**Figure 2:**
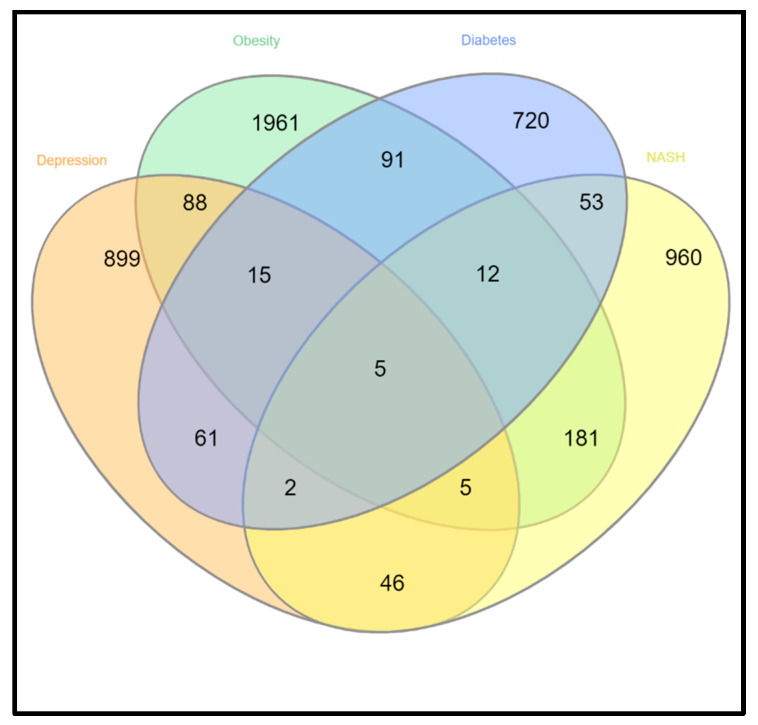
Venn diagram showing the number of common genes among all the experimental datasets.

**Figure 3:**
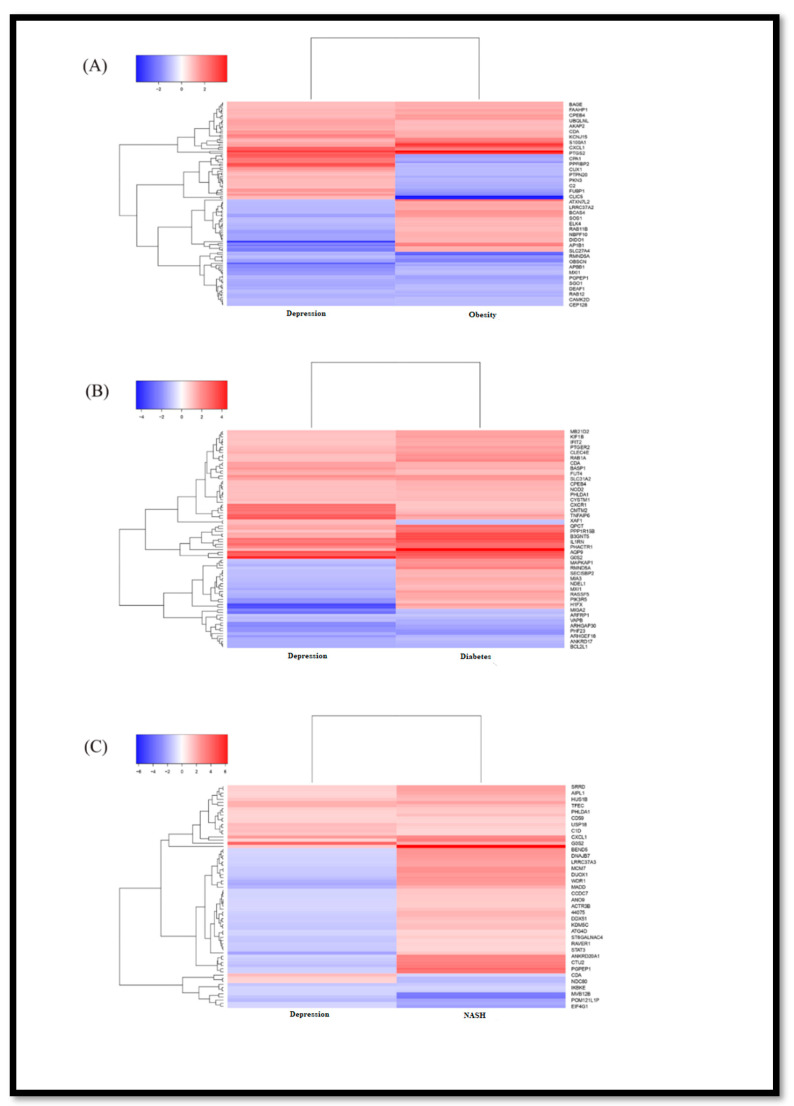
The heatmaps to identify up- and down-regulated genes from co-expression analysis in depression and obesity (**A**), depression and diabetes (**B**), depression and NASH (**C**) datasets.

We studied selected, independent, microarray datasets, and after normalization of the dataset GSE58430 (depression) showed 1092 DEGs (345 up,747 down), the dataset for obesity, GSE128021, contained 2422 DEGs (1190 up and 1230 down), the dataset for diabetes, GSE43950, showed 1179 DEGs (848 up, 331 down), the dataset for NASH, GSE46300, showed 1577 DEGs (1227 up, 350 down), which are listed in Supplementary Data S1–S4, respectively. Significantly expressed upregulated and downregulated DEGs for individual datasets were plotted in volcano plots as shown in Figure 1. In Figure 2, the Venn diagram made in R represents the number of co-expressed DEGs among the individual datasets. We found that depression datasets share a number of differentially expressed genes with the datasets of obesity (113 genes), diabetes (83 genes) and NASH (71 genes). We tried to emphasize the co-expressed genes among the associated disease between depression and metabolic disease groups. Comprehensive bioinformatics was performed to determine the independent DEGs across all the datasets. Enrichment pathway analysis was also performed to identify the pathways between the co-expressed DEGs. Notably we found five genes to be dysregulated among all the selected datasets namely, PRDM2, CXCL1, PHLDA1, DIDO1, CDA. Figure 3A–C represents the heatmaps of commonly altered genes among depression and obesity, diabetes and NASH, respectively to identify the expression pattern according to their changes in log fold values.

### 3.2. KEGG Pathway Analysis

**Figure 4:**
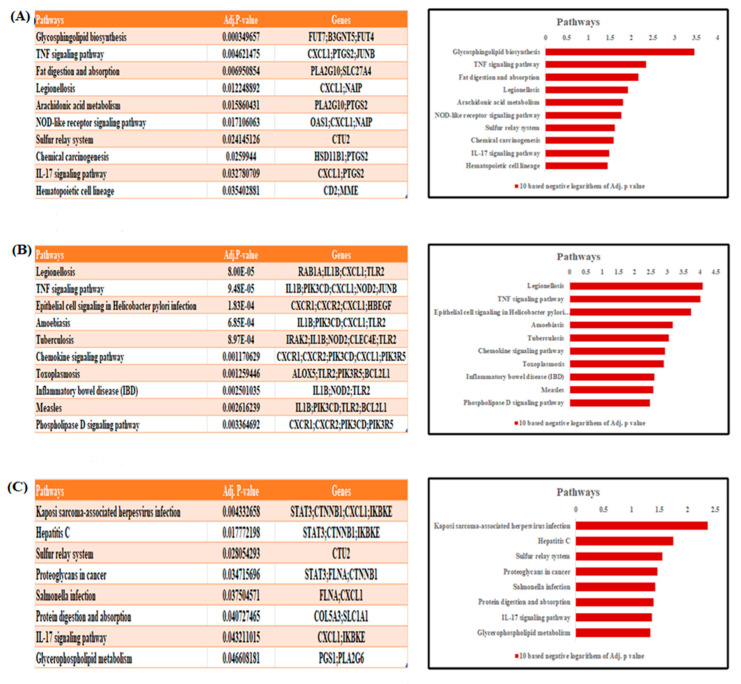
Pathway analysis identifying the top 10 most significant KEGG pathways related to the onset of depression and the metabolic disorders, revealed by the common differentially expressed genes. This includes (**A**) obesity (**B**) diabetes (**C**) NASH.

Enriched pathways are a series of molecular mechanisms and their interconnections. To identify a disease network pathway that plays an important role we employed commonly altered DEGs in depression and three other metabolic diseases to identify the biologically active pathways. After applying several statistical analyses, the ten most significantly enriched pathways in KEGG are shown in Figure 4. KEGG pathways associated with depression and diabetes or NASH are mostly related to infectious diseases including legionellosis, hepatitis C tuberculosis, measles, and salmonellosis. Notably, some of the pathways affect TNF and IL–17 signaling pathways, phospholipase D signaling pathways, protein digestion and absorption, and glycophospholipid metabolism. Among all the significantly enriched pathways, the TNF signaling pathway is commonly found in obesity and diabetes, whereas the IL–17 signaling pathway and sulfur relay system are common in both obesity and NASH.

### 3.3. Gene Ontological Pathway Analysis

Ontology pathways includes all the altered biological and disease pathways and their relationship in the disease domain. We utilized the commonly altered genes associated with depression and three other metabolic disease-related datasets and found the significantly enriched ontology pathways in the DAVID database [35]. Table 3, Table 4 and Table 5 showed the ten most significant ontology pathways linked with biological pathways, molecular functions and cellular components, respectively, in obesity, diabetes and NASH.

The significant pathways associated with the common DEGs in depression and obesity are the type I interferon signaling pathway, cytokine-mediated signaling pathway, and insulin secretion involved in the cellular response to glucose stimulus. For common DEGs in depression and diabetes, we found interleukin-1 receptor binding, peptidoglycan binding, phosphatidylinositol-4,5-bisphosphate 3-kinase activity, phosphatidylinositol bisphosphate kinase activity, chemokine receptor activity, inflammatory response, and cytokine-mediated signaling pathway. For common DEGs in depression and NASH, we found the cardiolipin biosynthetic pathway, phosphatidylglycerol biosynthetic pathway, thyroid hormone pathway and cardiolipin metabolic pathway. Mostly, these pathways are associated with lipid biosynthetic pathways and inflammation pathways.

### 3.4. PPI Interactions

**Figure 5:**
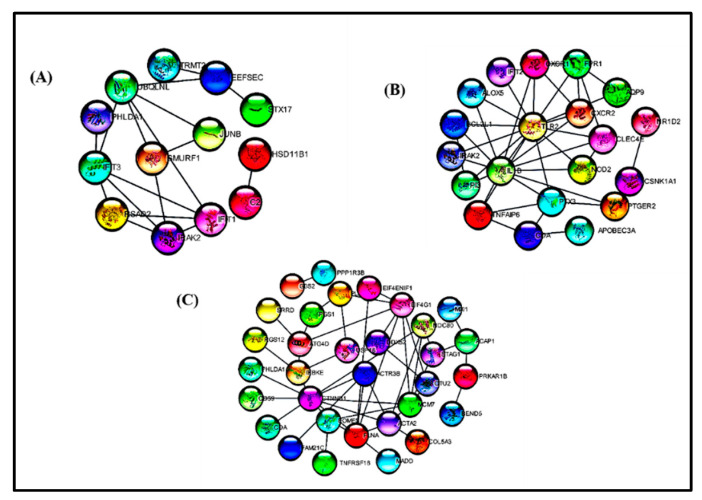
Protein–protein interactions between the common DEGs in the experimental datasets (**A**) depression and obesity, (**B**) depression and NASH and (**C**) depression and diabetes.

A protein–protein interaction helps to establish the connection between two specific proteins to build up functional biochemical networks for biological functions in the cells. We constructed protein–protein interaction networks with commonly altered genes associated with depression and the other three metabolic diseases with the STRING database [25], and visualized clusters consisting of hub genes discovered in each experimental dataset using the cytoscape software, as shown in Figure 5. In the CytoHubba plugin of cytoscape software altogether 29 hub genes were discovered, having >10 degrees and maximum nodes and neighborhood components. Among the 29 hub genes, 5 hub genes were differentially expressed across all the experimental datasets including depression, obesity, diabetes and NASH.

### 3.5. MCODE

**Figure 6:**
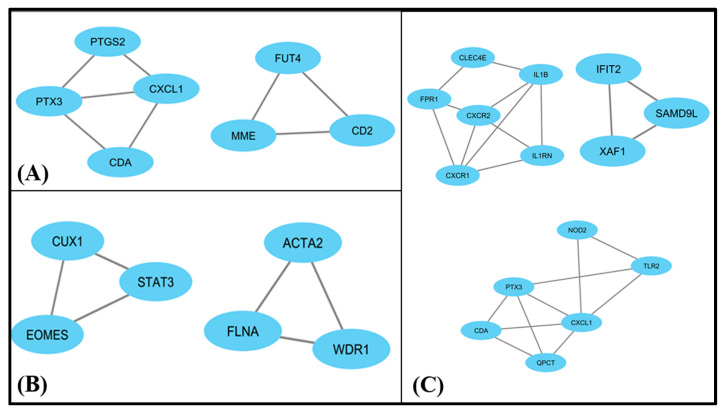
(**A**) depicts two clusters among the common DEGs in obesity and depression datasets; (**B**) shows two clusters with 3 nodes each among the common DEGs in NASH and depression datasets, and (**C**) shows three clusters with multiple nodes among the common DEGs in diabetes and depression datasets.

Molecular complex detection was used to find the most significant functional interactions between proteins modular clusters from the dense PPI network region. PPI networks in obesity and depression datasets showed two clusters, as shown in Figure 6. Cluster 1 contains four nodes with PTGS2, PTX3, CDA, CXCL1 genes and cluster 2 contains three nodes with FUT4, MME and CD2 genes. Simultaneously, two clusters were obtained from the PPI interactions for depression and NASH datasets, and both contain only three nodes. One contains CUX1, STAT3, EOMES and the other one contains ACTA2, FLNA and WDR1. For diabetes and depression datasets there are three clusters with multiple nodes. Cluster 1 contains six nodes with CXCR1, IL1RN, CXCR2, IL1B, FPR1, CLEC4E, with 10 edges and four density nodes. Cluster 2 contains NOD2, CXCL1, QPCT, CDA, PTX3, TLR2 with ten edges and four density nodes. Cluster 3 has only three nodes, with XAF1, IFIT2, SAMD9L genes.

### 3.6. TFs Prediction by iRegulon

To identify gene regulatory networks we used iRegulon, a computational method used to discover various transcription factors and their target genes, which in turn can identify a set of co-expressed genes. In the common genes between depression and obesity, we found five TFs. Among them FOXN4, SPDEF, NKX2-1 have 12, 10 and 9 targets, respectively, and MECOM and HDAC2 have 7 targets. MECOM is identified as an oncogene, stimulating cell proliferation and development, whereas HDAC2 is an important gene that belongs to the histone acetylase family and plays a major part in cell-cycle progression and developmental events. Among the common genes of depression and diabetes, there are four TFs, namely, BCL3, MXI1, GMEB2, NFKB1. BCL3 and GMEB2 have 31 targets. BCL3 is a proto-oncogene, and it acts as a transcription coactivator and activates GMEB2 through NFKB dimers, which increases sensitivity to lower the concentrations of glucocorticoids. The common DEGs of depression and NASH have four TFs, i.e., ATF, SRF, SREBF1, SCRT2. SREBF1 and SRF have the highest targets with 18 and 14, respectively. SREBF1 promotes cholesterol biosynthesis and lipid homeostasis. On the other hand, the SRF transcription factor is identified as a novel upstream mediator of stress.

### 3.7. Validation by OMIM Disease and dbGap

**Figure 7:**
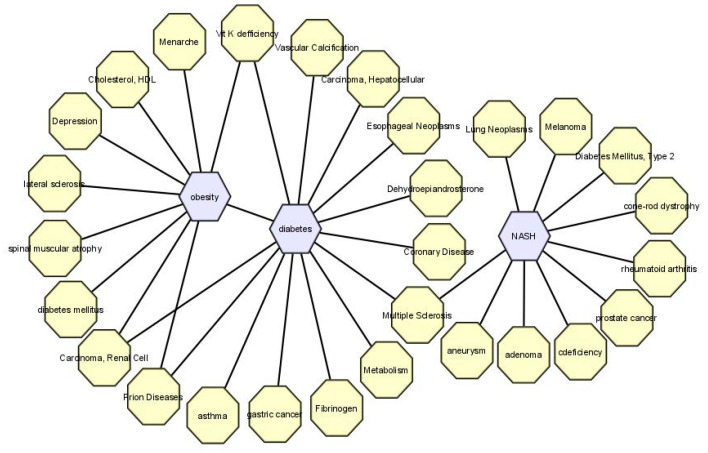
Gene disease association from OMIM disease database.

For validation of the DEGs obtained from PPI interactions and significantly enriched pathways in the depression dataset, we compared all the associated genes with the dbGaP, OMIM Disease and OMIM Expanded databases using only differentially expressed genes of depression so that we could potentiate the association between metabolic diseases and depression. After the analysis of all the related diseases, we found our selected three diseases, i.e., obesity, diabetes and NASH, among the list of all metabolic diseases which are associated with depression, as depicted in Figure 7.

### 3.8. Validation of the Commonly Expressed DEGs with Other Depression-Related Datasets

Our results show that PRDM2 is upregulated in two other datasets while it is downregulated in another. CXCL1 is downregulated in all other datasets including the dataset we analyzed. PHLDA1 is upregulated in all the datasets, which is inconsistent with our result. Both DIDO1 and CDA are upregulated in our dataset which is consistent with two other datasets, but downregulated in the other two, as referred to in Table 6.

## 4. Discussion

In our study, all the disease conditions selected are interconnected. We first hypothesized that the occurrence of one disease type may increase the chance of occurrence of the remaining three. The cellular origins of microarray datasets compared in our analysis are very diverse. Therefore, it cannot be ruled out that the expression values of a specific gene could be influenced by heterogeneous cell lines. The effect of these diseases is tissue-specific and dominant in those specific cell lines, hence, to mask the complications arising from the different experimental systems, compound traits or ethnicity employed in the original datasets, we normalized the gene expression data using Z-score transformation.

In this study, we investigated the effect of depression on its comorbidity in three metabolic diseases, i.e., obesity, diabetes and NASH. Hence, we compared the co-expression of the up- and down-regulated genes on MDD patients with that of obese, diabetic and NASH patients. To identify the commonly altered genes and their significantly enriched signaling pathways between depression and metabolic diseases, our study aimed to analyze the GEO microarray datasets on patients with depression, obesity, diabetes and NASH and their control datasets.

Our gene expression analysis suggested that depression and these metabolic diseases share a good number of commonly dysregulated genes. This indicates that depression has a strong influence on metabolic diseases [36]. The HPA axis is affected in depression, as a result it promotes hyperphagia which has a direct effect on metabolism. On the contrary, metabolic diseases cannot affect the HPA axis, so we cannot conclude that depression has a strong influence on metabolic disease or vice versa. To elucidate the molecular aspects of the regulatory network, we focused on pathway-based analysis, which is considered a new approach, to elucidate the molecular disease-network complexities related to the onset of depression. We found significantly enriched KEGG pathways for commonly dysregulated genes in depression related to each metabolic disease. These identified pathways strongly suggest that depression acts as a pivotal risk factor aggravating several metabolic disorders. Notably, gene expression ontologies and protein–protein interactions of commonly altered genes in depression and metabolic diseases revealed that depression plays a vital role in the prognosis of several metabolic diseases. We obtained two clusters among the common DEGs of both depression and obesity, and depression and diabetes, whereas three clusters were obtained in depression and NASH. PTX, CDA, and CXCL were present in the clusters among depression and obesity, and depression and NASH.

We verified our results with the gold benchmark database and found several genes play significant roles in both depression and other metabolic diseases. We collected disease names from the OMIM diseases, OMIM expanded, dbGap database using the commonly expressed dysregulated gene sets obtained from co-expression results in depression and metabolic diseases. Interestingly we identified the three metabolic diseases in the OMIM disease databases which coincide with the metabolic diseases we selected for the analyses. Moreover, the identified genes were significantly matched with the data found in these datasets.

Additionally, we investigated five genes, PRDM2, CXCL1, PHLDA1, DIDO1, CDA, which were commonly expressed in all datasets including depression, obesity, diabetes, and NASH. In our results, we found that PRDM2 is downregulated in NASH and depression datasets whereas it is upregulated in diabetes and obesity. On the other hand, except for obesity, all other datasets showed upregulation of CXCL1. PHLDA1 is upregulated in all the datasets except obesity. DIDO1 is only upregulated in the NASH dataset, and lastly, CDA is downregulated in obesity and NASH and upregulated in the other two datasets. Supplementary Data S5 shows the expression levels of these five commonly altered genes in each experimental dataset. To validate these commonly expressed DEGs among our experimental datasets, we compared their expression with other microarray GEO datasets related to depression in humans to ensure that the expression of these five genes were significantly related to the onset of depression. The expression level of these DEGs varied in all datasets related to depression due to the variation in the diverse cellular origins and the sample sizes. Our results are also in accordance with previously reported studies that suggest the same physiological role of these genes regarding depression and the metabolic diseases which we have obtained from our result. W. Fang et al. suggested that chromosomal deletion in the RIZ locus in PRDM2 may contribute to hepatocellular carcinoma which concomitantly can affect the progression of NASH [37,38]. The role of PRDM2 in high-fat induced obesity has been reported by Xie et al. via AKT transcription and AKT phosphorylation pathways [39]. Another chemokine ligand, CXCL1, plays a pivotal role in the pathogenesis of depressive disorder via inflammatory cytokines, as suggested by Hui et al. and Ślusarczyk et al. [40,41]. The CXCL1 chemokine gradient is also considered as a key factor to mediate obesity-dependent tumor-growth promotion according to the study by Zhang et al. [42]. In another study, CXCL1 was identified as one of the important markers of islets dysfunction and failure in T2DM [43]. CXCL1 is also elevated in NASH due to neutrophil infiltration [44]. Another important commonly dysregulated gene in our study, PHLDA1, is responsible for postpartum depression [45]. Loss of PHLDA1 also causes obesity, insulin resistance and NASH, while regulating lipogenesis, as suggested by Basseri et al. [46]. On the other hand, DIDO1 is associated with depression by affecting thyroid hormone levels [47]. Lastly, another co-expressed gene we found in our study was CDA, whose gene deficiency can lead to replicative stress as suggested by Farnces et al. [48].

It should be noted as a limitation of our study was that our report analyzed publicly available independent datasets which contain different types of cells and sample sizes. Consequently, it should also be considered that different cell lines have different expression values for a specific gene. Hence, this area should be further explored with in vivo and in vitro analysis for depression-related dysfunction, particularly with the brain region, which may strengthen the conclusion.

## 5. Conclusions

In this study, we considered gene expression (GEO) microarray data from depression and obesity, diabetes, NASH and control datasets to analyze and investigate the genetic links between depression and its effects on metabolic disorders. We analyzed gene expression, constructed gene–disease association networks, identified signaling and ontological pathways, analyzed protein–protein interaction networks, validated our results from the OMIM disease database, and finally compared the differentially co-expressed genes’ expression with other GEO datasets. The outcome of our study confirmed that depression may exert a strong influence on metabolic disorders. Moreover, we identified five potential DEGs that are co-expressed in all the experimental datasets in depression and metabolic diseases. We believe our study will be useful for making more accurate disease predictions and identifying potentially better therapeutic approaches.

## Figures and Tables

**Table 1:** Summarized description of the datasets used for experimental analysis.

SL	Disease Name	GEO Accession	Sample		Platform
			Healthy	Disease	
1	Depression	GSE58430	6	6	GPL14550
3	Obesity	GSE128021	3	3	GPL10558
2	Diabetes	GSE43950	5	4	GPL10379
4	NASH	GSE43600	8	10	GPL10558

**Table 2:** Validation of the common DEGs used for experimental analysis with related and unrelated diseases.

Disease Name	GEO Accession	Disease Name	GEO Accession	% of Upregulated Common DEGs	% of Down Regulated Common DEGs
Depression	GSE58430	Obesity	GSE128021	3.5	4.6
		Diabetes	GSE43950	5.1	3.9
		NASH	GSE43600	3.4	2.8
Influenza	GSE111449	Obesity	GSE128021	1.2	1.0
		Diabetes	GSE43950	0.7	0.6
		NASH	GSE46300	1.6	0.9

**Table 3:** GO pathways associated with significantly common DEGs in depression and obesity.

	GO Pathways		Adj *p*-Value	Genes
**Molecular functions**	RNA polymerase II regulatory-region sequence-specific DNA binding	(GO:0000977)	0.000059725	ELK4;CUX1;MAX;DEAF1;MAZ;TFEC;HSF1;MXI1;PRDM2;NKX2-5;JUNB
	alpha-(1->3)-fucosyltransferase activity	(GO:0046920)	0.000866475	FUT7;FUT4
	G-protein-activated inward rectifier potassium channel activity	(GO:0015467)	0.001382294	KCNJ15;KCNJ2
	fucosyltransferase activity	(GO:0008417)	0.002369586	FUT7;FUT4
	titin binding	(GO:0031432)	0.002754338	CAMK2D;OBSCN
**Biological pathways**	cellular response to type I interferon	(GO:0071357)	0.000033205	RSAD2;OAS1;IFIT1;XAF1;IFIT3
	type I interferon signaling pathway	(GO:0060337)	0.000033205	RSAD2;OAS1;IFIT1;XAF1;IFIT3
	cytokine-mediated signaling pathway	(GO:0019221)	0.000244271	CAMK2D;RSAD2;OAS1;IRAK2;TNFRSF10C;CXCL1;IFIT1;XAF1;SOS1;PTGS2;JUNB;IFIT3
	insulin secretion involved in cellular response to glucose stimulus	(GO:0035773)	0.000467629	PTPRN;RAB11B
	L-fucose metabolic process	(GO:0042354)	0.001109929	FUT7;FUT4
**Cellular components**	Golgi subcompartment	(GO:0098791)	0.001591532	SLC35A2;FUT7;CUX1;CDH1;B3GNT5;RAB12;AP1B1;MAN1C1;FUT4
	spindle pole	(GO:0000922)	0.003189301	SGO1;STAG2;HSF1;CEP128
	tertiary granule lumen	(GO:1904724)	0.003721186	CDA;CXCL1;PTX3
	nuclear transcription-factor complex	(GO:0044798)	0.007595372	MAX;MXI1;NKX2-5
	mitotic spindle pole	(GO:0097431)	0.008720051	STAG2;HSF1

**Table 4:** GO pathways associated with significantly common DEGs in depression and diabetes.

GO Pathways			Adj *p*-Value	Genes
**Molecular functions**	interleukin-1 receptor binding	(GO:0005149)	0.001725028	IL1RN;IL1B
	peptidoglycan binding	(GO:0042834)	0.001966159	NOD2;TLR2
	phosphatidylinositol-4,5-bisphosphate 3-kinase activity	(GO:0046934)	0.002844067	PIK3CD;HBEGF;PIK3R5
	phosphatidylinositol bisphosphate kinase activity	(GO:0052813)	0.00321474	PIK3CD;HBEGF;PIK3R5
	chemokine receptor activity	(GO:0004950)	0.003394807	CXCR1;CXCR2
**Biological pathways**	neutrophil degranulation	(GO:0043312)	0.0000000834	CDA;TNFAIP6;MME;FPR1;CXCL1;CXCR1;ALOX5;QPCT;CXCR2;CYSTM1;PTX3;S100A11;TLR2
	neutrophil activation involved in immune response	(GO:0002283)	0.0000000918	CDA;TNFAIP6;MME;FPR1;CXCL1;CXCR1;ALOX5;QPCT;CXCR2;CYSTM1;PTX3;S100A11;TLR2
	neutrophil-mediated immunity	(GO:0002446)	0.0000001009	CDA;TNFAIP6;MME;FPR1;CXCL1;CXCR1;ALOX5;QPCT;CXCR2;CYSTM1;PTX3;S100A11;TLR2
	inflammatory response	(GO:0006954)	0.000001012	TNFAIP6;IL1B;PTGER2;CXCR2;FPR1;PIK3CD;CXCL1;PTX3;NOD2
	cytokine-mediated signaling pathway	(GO:0019221)	0.0000113203	IL1RN;IRAK2;IL1B;ALOX5;FPR1;PIK3CD;CXCL1;NOD2;XAF1;JUNB;IFIT2;BCL2L1
**Cellular components**	tertiary granule lumen	(GO:1904724)	0.00000322	CDA;TNFAIP6;QPCT;CXCL1;PTX3
	tertiary granule	(GO:0070820)	0.00000540	CDA;TNFAIP6;QPCT;FPR1;CXCL1;CYSTM1;PTX3
	ficolin-1-rich granule	(GO:0101002)	0.001014794	CDA;TNFAIP6;ALOX5;QPCT;FPR1
	ficolin-1-rich granule lumen	(GO:1904813)	0.001721089	CDA;TNFAIP6;ALOX5;QPCT
	secretory granule lumen	(GO:0034774)	0.002052377	CDA;ALOX5;QPCT;CXCL1;PTX3;S100A11

**Table 5:** GO pathways associated with significantly common DEGs in depression and NASH.

GO Pathways			Adj *p*-Value	Genes
**Molecular functions**	ATP-dependent helicase activity	(GO:0008026)	0.003438361	MCM7;DDX51;DDX52
	RNA polymerase II transcription-factor binding	(GO:0001085)	0.009156092	EOMES;STAT3;CTNNB1
	RNA polymerase II activating transcription-factor binding	(GO:0001102)	0.012116708	EOMES;CTNNB1
	inorganic anion transmembrane transporter activity	(GO:0015103)	0.012614773	SLC22A4;SLC1A1
	small GTPase binding	(GO:0031267)	0.021109349	ACAP1;FLNA
**Biological Pathways**	cardiolipin biosynthetic process	(GO:0032049)	3.43 × 10^−4^ 0.000343	PGS1;PLA2G6
	phosphatidylglycerol biosynthetic process	(GO:0006655)	5.49 × 10^−4^ 0.000549	PGS1;PLA2G6
	thyroid-hormone generation	(GO:0006590)	5.49 × 10^−4^ 0.000549	DUOX1;DIDO1
	cardiolipin metabolic process	(GO:0032048)	9.45 × 10^−4^ 0.000945	PGS1;PLA2G6
	cytoplasmic sequestering of protein	(GO:0051220)	0.00126624	MXI1;FLNA
**Cellular functions**	nucleolus	(GO:0005730)	0.010004394	C1D;MXI1;FLNA;DDX51;RGS12;DDX52;PHLDA1
	RNA polymerase II transcription-factor complex	(GO:0090575)	0.015449845	MXI1;STAT3;CTNNB1
	tertiary granule lumen	(GO:1904724)	0.016344963	CDA;CXCL1
	tertiary granule	(GO:0070820)	0.020605435	CDA;CXCL1;CD59
	THO complex part of transcription export complex	(GO:0000445)	0.021114315	THOC3

**Table 6:** Expression of the commonly expressed DEGs with other depression-related GEO datasets.

GEO Accession	Sample Type	PRDM2	CXCL1	PHLDA1	DIDO1	CDA
**GSE14922**	Control vs. cortisol secreting adenoma	Down regulated	Down regulated	Down regulated	Up regulated	Up regulated
**GSE109857**	Control vs. glioma	Up regulated	Down regulated	Down regulated	Down regulated	Down regulated
**GSE114852**	Control vs. depression	absent	Down regulated	Down regulated	Up regulated	Down regulated
**GSE32280**	Control vs. depression	Up regulated	Down regulated	Down regulated	Down regulated	Up regulated
**GSE58430**	Control vs. depression	Down regulated	Down regulated	Up regulated	Down regulated	Up regulated

## Data Availability

The data that support this study are available within the reference and its supplementary data files or available from the authors upon request.

## Supplementary Materials

The following are available online at https://www.mdpi.com/article/10.3390/life11111203/s1, Supplementary Data S1. Upregulated and downregulated DEGs from dataset GSE58430; Supplementary Data S2. Upregulated and downregulated DEGs from dataset GSE128021; Supplementary Data S3. Upregulated and downregulated DEGs from dataset GSE43950; Supplementary Data S4. Upregulated and downregulated DEGs from dataset GSE46300; Supplementary Data S5. Expression level of these five commonly altered genes in each experimental dataset.

## Abbreviations

DEGs	Differentially Expressed Genes
Log FC	Log Fold Change
GO	Gene Ontology
GEO	Gene Expression Omnibus
KEGG	Kyoto Encyclopedia of Genes and Genomes
PPI	Protein–Protein Interaction
DAVID	Database for Annotation, Visualization, and Integrated Discovery
NASH	Nonalcoholic Steatohepatitis
MDD	Major Depressive Disorder
NCBI	National Center for Biotechnology Information
OMIM	Online Mendelian Inheritance in Man
HPA	Hypothalamic–pituitary–adrenal
CRH	Corticotrophin-releasing hormone
